# Is It a Good Time to Be a Pharmacist in the US?

**DOI:** 10.3390/pharmacy6030061

**Published:** 2018-07-03

**Authors:** Surrey M. Walton, Henri R. Manasse

**Affiliations:** Department of Pharmacy Systems Outcomes and Policy, UIC College of Pharmacy, 833 S. Wood Street (871), Room 287, College of Pharmacy, Chicago, IL 60612, USA; hrmjr@uic.edu

**Keywords:** pharmacists, labor market, autonomy

## Abstract

The labor market for pharmacists in the United States has seen significant dynamics over the past couple of decades in both demand and supply. The purpose of this brief editorial is to discuss some key concerns for pharmacists in the current labor market and over the next decade. A key issue in evaluating how pharmacists will fare in moving forward into expanded clinical roles and functions will be the degree to which they expand their professional autonomy.

The labor market for pharmacists in the United States has seen significant dynamics over the past couple of decades in both demand and supply. In particular, there have been substantial and ongoing increases in the number of graduates from pharmacy degree programs. These have been fueled by both larger enrollments in existing programs and the emergence of an almost doubling programs across the US since 1990 [1]. In the midst of this trend, two of the most recent sets of forecasts related to the demand of pharmacists relative to their supply stand out. First, the National Center for Health Workforce Analysis, sponsored by the Health Resources and Services Administration, projected that between 2012 and 2025 the pharmacist supply, adjusting for new entrants as well as exit from the labor force, would grow by 35% while demand would only increase by 16% [2]. Second, the Bureau of Labor Statistics (BLS) estimates only a 6% increase in employment of pharmacists between 2016 and 2026 [3]. Although the BLS estimates are based on the numbers of jobs rather than numbers of people in the workforce, the difference in rates is cause for concern since the BLS estimates an increase of 17,400 jobs over 10 years, while there are currently over 14,500 new graduates per year [4]. Together these forecasts suggest a tightening of the labor market over the next decade. However, it is important to understand that the accuracy of these kinds of forecasts can be impacted by general trends in the overall economy, attractiveness of other career areas, as well as changes in health coverage policies, state laws related to practice, and technological changes. Additionally, in assessing the future of the profession of pharmacy, there are other current market dynamics that are shaping the future nature of scope and focus of the work of pharmacists. For example, as pointed out recently by Lucinda Maine, the scope of training of pharmacists has expanded substantially along with an expansion of new professional opportunities [5]. Further, as acknowledged in the Health Resources & Services Administration (HRSA) forecasting report, greater integration of pharmacists into medical teams across a variety of potential settings, along with more advanced clinical roles, has the potential to substantially boost demand well beyond current expectations [2,6].

A key issue in evaluating how pharmacists will fare in moving forward into expanded clinical roles and functions will be the degree to which they expand their professional autonomy. A key component of a profession, one that separates the notion of a profession versus other occupations, is autonomy. That is, the degree to which the pharmacist can make clinical decisions based on their unique knowledge and skills without ‘asking permission’, establishes their level of professional autonomy [7]. Similar evolution has been witnessed in the profession of nursing around the expanding role of the advanced nurse practitioner and associated autonomy from oversight by physicians. Beginning on 1 January 2018, for example, advanced nurse practitioners who meet required State of Illinois criteria, can practice without Collaborative Practice Agreements or ‘sign offs’ from physicians. Similar directions are being taken in most states in the profession of nursing.

Of natural consequence, autonomy rests on accountability, which often serves as a restraint to a profession in its pursuit of autonomy. In contrast to the evolution of nurse practitioners in the United States, pharmacists have remained conservative in their approach to autonomy. This manifests itself in the ongoing political philosophy of practice acts in pharmacy which typically rely on collaborative practice agreements, regulatory oversight and approval and other mechanisms by which autonomy may be curtailed. Currently, state practice acts often serve as a barrier to autonomy, and particularly in states with relatively strict practice acts. While pharmacists do need to balance accountability with autonomy, there appears to be ample room for them to broaden their scope of practice and as stated in a recent commencement speech at the University of Illinois Chicago “practice at the top of their education rather than at the top of their license” [8].

An important case study in evolutionary autonomy for pharmacists is the Veteran’s Administration (VA) system where pharmacists increasingly operate clinics in a number of chronic disease areas [9,10]. Such clinics focus on drug review and management of the patient to assure meeting clinical outcomes and reducing adverse drug effects. Underlying this development is the fact that the VA system operates under federal law which tends not to focus on practice as do state laws. Pharmacists in the VA are reviewed institutionally through Credentialing Committees and afforded different pay grades depending on their responsibilities and recognized credentials such as Board Certification by the Board of Pharmaceutical Specialties. Pharmacists are able to become board certified in eleven specialties which can then also lead to higher salaries.

It is often the case that in states with less restrictive practice laws, pharmacists are more likely to work in independent practices where they are afforded relatively high flexibility and greater autonomy in how they contribute to patient care. Hence, pharmacists are beginning to become part of physician practices. Again a point made by Lucinda Maine is that the expansion of pharmacists into medical practice has been a key demand driver in the wake of the enormous expansion of pharmacist graduates over the past 30 years [5].

As these trends continue, and as pharmacy education has been evolving to expand clinical knowledge and leadership skills, there will likely be further movements for expanded autonomy. Given the complexity and associated risks for drugs, biologicals, vaccines and contrast media, as well as increasing calls for safety and quality in the health care enterprise, the effective use of pharmacists would go a long way toward improving personal and population outcomes. Moreover, contemporary pharmacy graduates are looking for workplace involvement where their knowledge and skills can be effectively utilized. This has led to graduates being employed in hospitals, health systems and ambulatory clinics at a greater rate than in the traditional community chain or independent pharmacy settings.

For pharmacists to continue to expand their clinical impact, organized efforts at expanding autonomy and accountability to go along with enhanced skills and broadened scope of practice will be essential. Hence, there should be a focus on broadening scope of practice in practice laws, forming effective collaborations with other health professions, and pushing the health care system to adopt specific reimbursement policies for expert care delivered by pharmacists.

As with other professions, technological advances will play an increasing role in productivity as well as in expanding connections with patients. In addition to staying at the forefront of knowledge in how and when drugs work most effectively, efficiently and cost-effectively, pharmacists will have to continually work to stay at the forefront of tools for the delivery of their care services. Further, pharmacy education should include and promote leadership for enhanced professional autonomy and accountability in their students.

In conclusion, it is a good time to be a pharmacist in the US, but how good it is will depend on the ability of the profession to foster and strengthen autonomy in practice and to continue to expand their scope of practice and impact on patient outcomes. With that, influences on payment and reimbursement policy along with loosening of restrictions in pharmacy practice acts will be key, along with the continually expanding scope, quality, and depth of education and experience of current and future pharmacy professionals.
